# Associations Between Brain Structure and Dual Decline in Gait and Cognition

**DOI:** 10.1002/alz.089373

**Published:** 2025-01-09

**Authors:** Sadhani Karunarathna, Monique Breslin, Jane E Alty, Richard Beare, Taya Collyer, Velandai K Srikanth, Scott McDonald, Michele L Callisaya

**Affiliations:** ^1^ Menzies Institute for Medical Research, University of Tasmania, Hobart, TAS Australia; ^2^ Royal Hobart Hospital, Hobart, TAS Australia; ^3^ Wicking Dementia Research and Education Centre, University of Tasmania, Hobart, TAS Australia; ^4^ School of Medicine, University of Tasmania, Hobart, TAS Australia; ^5^ National Centre for Healthy Ageing, Melbourne, VIC Australia; ^6^ Murdoch Children’s Research Institute, Royal Children’s Hospital, Melbourne, VIC Australia; ^7^ Peninsula Clinical School, Central Clinical School, Monash University, Melbourne, VIC Australia; ^8^ Frankston Hospital, Peninsula Health, Melbourne, VIC Australia

## Abstract

**Background:**

Dual decline in gait and cognition is associated with an increased risk of dementia, with combined gait and memory decline exhibiting the strongest association. However, little is known about the underlying brain correlates. Therefore, we aimed to examine the associations between measures of brain structure and dual decline in gait and cognition across several cognitive domains.

**Method:**

Participants over 60 years were randomly selected from the electoral roll into the Tasmanian Study of Cognition and Gait. We used baseline MRI and three serial gait speed and cognitive assessments (memory, processing speed‐attention, verbal fluency) performed on average 2.5 years apart. Participants were classified into four groups depending on tertiles of annual decline in gait speed and each cognitive measure (non‐decliners, gait‐only decliners, cognition‐only decliners, and dual‐decliners). Dual decliners were defined as the participants belonging to the highest tertile of annual decline in both gait speed and each of the cognitive measures. Multinomial logistic regression was used to examine the associations of baseline brain volumes (gray matter, white matter, and hippocampal) and cerebral small vessel disease (white matter hyperintensities, subcortical infarcts, enlarged perivascular spaces, and microbleeds) with dual gait and cognitive decline for each cognitive domain.

**Result:**

The mean age of participants was 70.9 ± SD 6.7 years (n = 267). Lower baseline gray and white matter volume and higher white matter hyperintensity volume increased the risk of being a dual decliner in gait and both the memory and processing speed‐attention groups (all p < 0.05). Lower hippocampal volume (p = 0.047) was only associated with increased risk of being a dual decliner in the gait and memory group. No significant associations were found between MRI measures and the gait and verbal fluency dual decliners.

**Conclusion:**

Neurodegenerative pathology and white matter hyperintensities are involved in dual decline in gait and both memory and processing speed‐attention domains. Smaller hippocampal volume may also contribute to dual decline in gait and memory, but not dual decline in gait and other cognitive domains.

